# HGF/c-Met: A Key Promoter in Liver Regeneration

**DOI:** 10.3389/fphar.2022.808855

**Published:** 2022-03-17

**Authors:** Yang Zhao, Wenling Ye, Yan-Dong Wang, Wei-Dong Chen

**Affiliations:** ^1^ Key Laboratory of Receptors-Mediated Gene Regulation, The People’s Hospital of Hebi, School of Medicine, Henan University, Kaifeng, China; ^2^ State Key Laboratory of Chemical Resource Engineering, College of Life Science and Technology, Beijing University of Chemical Technology, Beijing, China

**Keywords:** HGF, c-Met, liver regeneration, uPA, cytokine

## Abstract

Hepatocyte growth factor (HGF) is a peptide-containing multifunctional cytokine that acts on various epithelial cells to regulate cell growth, movement and morphogenesis, and tissue regeneration of injured organs. HGF is sequestered by heparin-like protein in its inactive form and is widespread in the extracellular matrix of most tissues. When the liver loses its average mass, volume, or physiological and biochemical functions due to various reasons, HGF binds to its specific receptor c-Met (cellular mesenchymal-epithelial transition) and transmits the signals into the cells, and triggers the intrinsic kinase activity of c-Met. The downstream cascades of HGF/c-Met include JAK/STAT3, PI3K/Akt/NF-κB, and Ras/Raf pathways, affecting cell proliferation, growth, and survival. HGF has important clinical significance for liver fibrosis, hepatocyte regeneration after inflammation, and liver regeneration after transplantation. And the development of HGF as a biological drug for regenerative therapy of diseases, that is, using recombinant human HGF protein to treat disorders in clinical trials, is underway. This review summarizes the recent findings of the HGF/c-Met signaling functions in liver regeneration.

## Introduction

Hepatocyte growth factor (HGF) is secreted by mesenchymal cells and is expressed in different type cells such as hepatic stellate cells (HSC), vascular endothelial cells (ECs), and Kupffer cells (KCs). HGF is a specific ligand for the tyrosine kinase receptor c-Met (Miyazawa et al., 1989; Matsumoto and Nakamura, 1992; Trusolino et al., 2010). The HGF/c-Met pathway plays a crucial role in the protection and regeneration of tissues. In various models built for injury and disease, HGF promotes cell survival and tissue regeneration, and improves chronic inflammation and fibrosis (Michalopoulos and Khan, 2005; Jangphattananont et al., 2019). Simultaneously, HGF is always acted as a potent inducer of lymphangiogenesis, angiogenesis, and tumor growth through promoting tumor cell invasion and metastasis (Cienfuegos et al., 2014; Wang et al., 2020).

In 1984, Russell et al. identified and purified HGF in rat platelets (Nakamura et al., 1984; Russell et al., 1984; Nakamura et al., 1987), and Gohda et al. purified human HGF from the plasma of fulminant hepatic failure patients in 1988 (Gohda et al., 1988). In 1989, Nakamura et al. cloned the cDNA of human HGF and clarified the primary structure of HGF, and identified HGF as a novel growth factor that has a unique structural characteristic (Miyazawa et al., 1989; Nakamura et al., 1989). Then, researchers gradually found that the fibroblast-derived factor scatters factor (SF) and HGF have the same structure and function. They finally confirmed that they are identical proteins encoded by a single gene (Stoker et al., 1987; Gherardi et al., 1989; Weidner et al., 1990; Naldini et al., 1991a; Shima et al., 1991; Weidner et al., 1991; Ahn et al., 2019).

**FIGURE 1:**
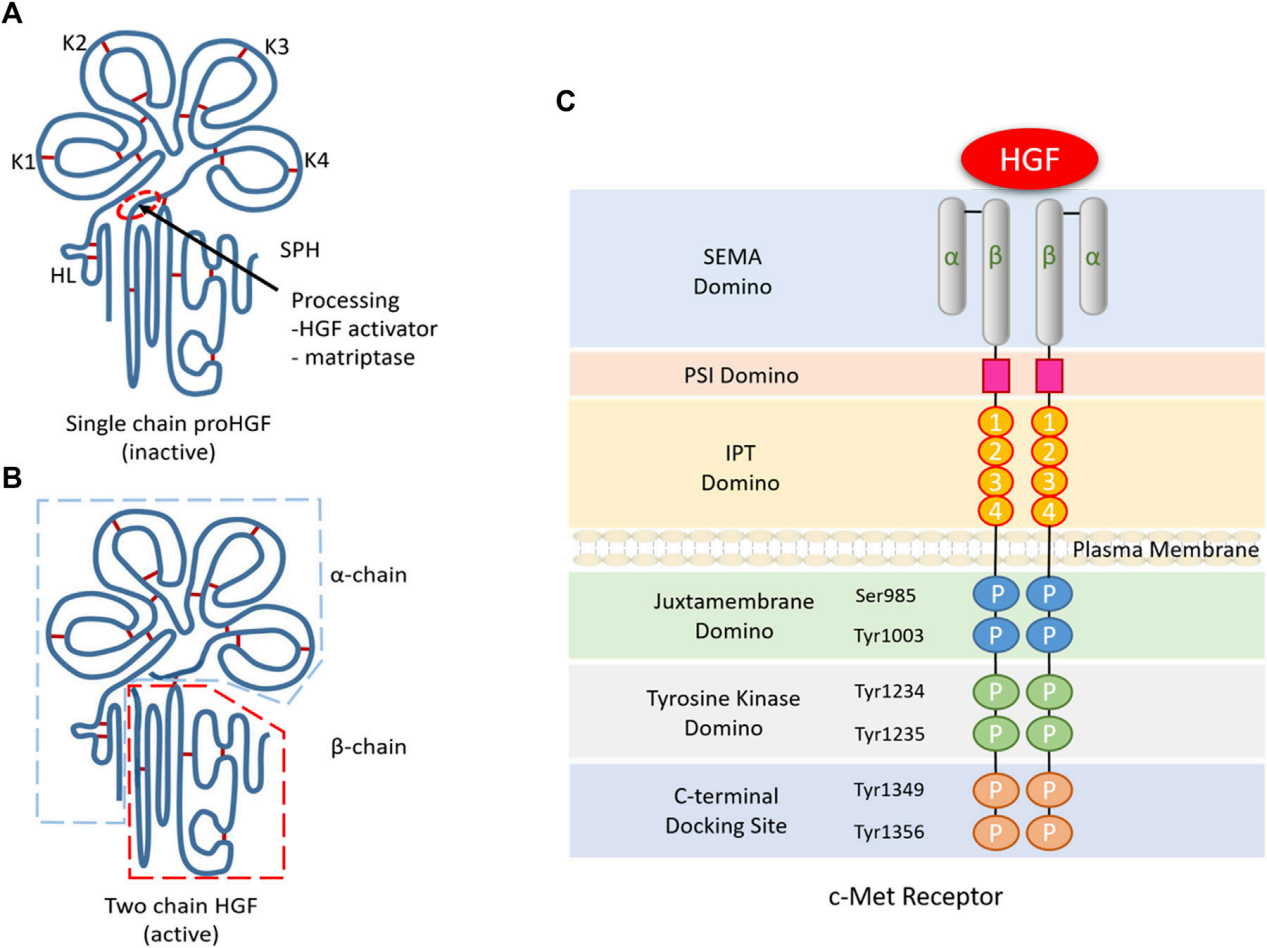
Structure and molecular signalling components of HGF/c-Met. **(A)**. The α-chain of HGF is composed of an N-terminal hairpin loop (HL) attached four kringle domains (K1-K4), and the β-chain is composed of a serine protease homology domain (SPH) lacking proteolytic activity. **(B)**. Active HGF is a heterodimeric molecule composed of an α-chain and a β-chain. **(C)**. C-Met consists of a small α-chain and a larger β-chain, including extracellular, transmembrane and intracellular domains. The extracellular domain contains a large semaphore protein (SEMA) domain where HGF binds to c-Met. After the SEMA domain, a plexin, semaphorin and integrin-rich (PSI) domain and four immunoglobulin/plexin/transcriptional factors (IPT) domains make up the rest of the extracellular domains. Intracellularly, the C-terminal tail of c-Met β-chain containing two phosphorylation sites (Ser985 and Tyr1003), two tyrosine residues (Tyr1234 and Tyr1235) and a multisubstrate docking site (Tyr1349 and Tyr1356).

The *HGF* gene is mapped on the long arm of chromosome 7 of q21.1, and alternative splicing results in multiple transcripts, at least one of which encodes an inactive pre-pro-HGF. Then, the pre-pro-HGF becomes pro-HGF after a cleavage process between Arg494 and Val495. Mesenchymal cells produced HGF, which is a specific paracrine factor. And it is secreted out of the cells in the precursor form (pro-HGF) and also activated by the proteolytic cleavage at the Arg-Val site (Naka et al., 1992; Mizuno et al., 1994). The active HGF consists of 697 or 692 amino acids as a heterodimeric composed of α-chain and β-chain (Kirchhofer et al., 2004). Four kringle domains constitute the α-chain, while serine protease-like structures constitute the β-chain (Figures 1A,B). HGF activators reported previously include serum proteases and cell membrane proteases, such as HGF activator (HGF-A), urokinase-type plasminogen activator (uPA), plasma kallikrein, coagulation factors XII and XI, metalloproteinases and heparin. HGF-A is the central protease responsible for activating pro-HGF in serum (Fajardo-Puerta et al., 2016). However, the studies in HGF-A knockout mice have shown that HGF-A gene deletion does not affect liver regeneration (Fukushima et al., 2018). In contrast, uPA plays a crucial role in HGF activation during liver regeneration (Roselli et al., 1998).

Unlike other growth factors and their receptors, HGF binds to the c-Met as a unique ligand (Bertotti and Comoglio, 2003; Birchmeier et al., 2003), which is also located on chromosome 7 (Bottaro et al., 1991; Naldini et al., 1991b; Dean et al., 1985). c-Met is a 190 kDa protein that contains a protein domain that binds to extracellular ligands, a transmembrane domain with tyrosine kinase activity, and a cytoplasmic domain. The c-Met has a 50 kDa β-chain and a 145 kDa α-chain for its mature form. The binding of HGF and c-Met can induce clustering of c-Met and phosphorylation of Y1234 and Y1235, and then phosphorylates Y1349 and Y1356 in the carboxy-terminal region (Figure 1C), which subsequently results in the adaptor molecule binding with it and transmitting downstream signals (Cecchi et al., 2012; Gherardi et al., 2012; Sakai et al., 2015). HGF has two individual MET-binding interfaces, which include a high affinity NK1 (N-terminal and first kringle domain) binding site and a low affinity β-chain binding site (Matsumoto et al., 2017).

The biological functions of HGF are extensive and diverse (Schmidt et al., 1995; Uehara et al., 1995; Samamé Pérez-Vargas et al., 2013; Cui, 2014; Maroun and Rowlands, 2014; Motoi et al., 2019; Mukai et al., 2020). In embryonic development, targeted destruction of HGF or c-Met could lead to mouse death *in utero* and damage the development of the liver and placenta (Schmidt et al., 1995; Uehara et al., 1995). Recombinant human HGF (rh-HGF) can significantly inhibit hepatocyte death and stabilize structural and vascular integrity in mice with acute liver failure (ALF) (Motoi et al., 2019). HGF receptor overexpression, mutation, amplification, or changes in its kinase activity is closely related to many different types of tumors (Samamé Pérez-Vargas et al., 2013; Cui, 2014; Maroun and Rowlands, 2014; Mukai et al., 2020).

HGF/c-Met signaling pathway is indispensable for liver regeneration and regular repair (Huh et al., 2004). Studies showed that the lack of c-Met leads to liver necrosis and jaundice, delays regeneration, and high mortality in rodents (Michalopoulos and DeFrances, 1997; Stolz et al., 1999; Borowiak et al., 2004; Huh et al., 2004). In the model of the normal rats and mice, injecting HGF into the portal vein caused hepatocyte proliferation and hepatomegaly (Liu et al., 1994a; Patijn et al., 1998). When recombinant human HGF-activator (rh-HGF-activator) was administered *via* the portal vein, compared with the control group, and the liver regeneration rate in the rh-HGF-activator-treated group was significantly higher (Kaibori et al., 2002). After partial hepatectomy (PHx), the tyrosine phosphorylation of c-Met occurs just in 5 min and increases gradually, then peaks at 60 min (Stolz et al., 1999). *In vitro*, the activated HGF binding to the c-Met drives the phosphorylation of c-Met tyrosine residues, then Wnt-independent nuclear translocation of β-catenin is induced by HGF (Monga et al., 2002). Subsequently, c-Met is internalized and degraded promptly by the ubiquitin-proteasome degradation pathway (Naka et al., 1993; Jeffers et al., 1997). In the initial stage of liver regeneration, HGF activates JAK/STAT3, PI3K/Akt/NF-κB, and Ras/Raf pathways *via* HGF/c-Met (Li et al., 2018a), and initiates cell proliferation.

In this review, we summarized the physiological and pathological mechanisms of the HGF/c-Met in liver regeneration. Specifically, we highlight the latest research on the HGF/c-Met axis, including the roles of non-coding RNAs in regulating of liver regeneration by HGF.

### Changes of HGF After PHx

PHx in mice or rats is a mature model for the study of the mechanism of liver regeneration. After PHx (>70%) or liver injury, rapid recovery of liver volume and function and strict regulation at the beginning and end stages are unique features of the liver (Cienfuegos et al., 2014). After PHx on mice or rats, the residual liver tissue recovered to the same level both in mass and volume as before in just 1 week. Both single-chain HGF and two-chain (active form) HGF are present in normal liver, and the single-chain HGF is the dominant form, while the expression of HGF mRNA is almost undetectable (Pediaditakis et al., 2001). After PHx, the level of HGF in the plasma has increased 10 to 20 times (Lindroos et al., 1991; Liu et al., 1994a; Patijn et al., 1998). According to the changes in endogenous HGF expression levels, liver regeneration can be divided into two stages. In phase I, the depletion phase, 0–3 h after PHx, HGF was derived from the transcription of *HGF* gene in KCs and ECs of normal liver, with a decrease in the level of both single-chain HGF (inactive form) and active two-chain HGF (active form) as characteristic (Maher, 1993). In phase II, the production phase, from 3 to 48 h or more after PHx, HGF was newly synthesized by ECs and HSCs and characterized by a significant increase in both single-chain and two-chain HGF levels. In addition, only active two-chain HGF was detected in plasma during the first 3 h after PHx (Lindroos et al., 1991; Pediaditakis et al., 2001). At the end of the liver regeneration process, TGF-β plays a role in the extracellular matrix (ECM) reconstruction. And with the ECM rebuilds, TGF-β binds to HGF, blocking its activation and restoring the hepatocytes to stasis (Kogure et al., 2000; Ichikawa et al., 2001).

### uPA Activation and HGF in Liver Regeneration

Another earliest biochemical change observed after PHx was the increased activity of uPA. Inactive pro-HGF was activated by uPA in the ECM (Shimizu et al., 2001). How do hemodynamic changes regulate the uPA activation is still unclear. However, uPA activation in such endothelial cells associated with the increased mechanical stress and turbulence have been documented (Sokabe et al., 2004).

We know that the microenvironment, including cells and ECM plays a crucial role in maintaining liver tissue homeostasis and regeneration (Fausto et al., 2006; Michalopoulos, 2010). The remodeling of ECM is accompanied by the renewal of many proteins. Urokinase is an activator for matrix remodeling and is detectable in tissues suffering wound healing and liver regeneration. After PHx, the uPA quantity remains relatively constant, but the uPA receptor (uPAR) dramatically increases after PHx, just beginning within 1 min. It enhances the activity of uPA in all remaining liver lobes (Mars et al., 1995; Kortlever and Bernards, 2006). Then, the uPAR is induced to activate the fibrinolytic cascade reaction, and the plasminogen is activated to plasmin within 15 min (Kim et al., 1997). Plasminase promotes the conversion of matrix metalloproteinases to active metalloproteinases at 30 min after PHx (Kim et al., 2000). Both metalloproteinase and plasmin play a role in matrix remodeling and the renewal of ECM. The remodeling of ECM triggers a signal impulse through integrin, which leads to activating the inactive HGF attached to the ECM into its active form. It causes local and systemic HGF receptor c-Met activation within 30 min to 1 h after PHx (Kim et al., 1997). So metalloproteinase and metalloproteinase tissue inhibitor levels are essential in regulating the release and activation of HGF during regeneration (Mohammed et al., 2005; Mohammed and Khokha, 2005).

A great deal of work focusing on the regulation of uPA activity in the initial stage of liver regeneration is underway. Recent reports show that the increased release of preformed HGF in the extracellular matrix during liver regeneration which supports liver regeneration, is due to the up-regulation of uPA by TLR3 (Stöß et al., 2020). And during hepatitis B virus infection, no matter in mouse liver tissues or human liver cell lines, HBx down-regulated expression of uPA by the uPA promoter epigenetic regulation, resulting in damage to liver regeneration (Park et al., 2013). Deletion of nuclear factor ImerC (NFI-C) leads to overexpression of plasminogen activator I (PAI-1) and subsequent inhibition of uPA activity and HGF signal, thus impairing hepatocyte proliferation (Edelmann et al., 2015). The hepatic hemodynamics changed immediately after PHx, and the portal vein pressure increased about three times theoretically (Marubashi et al., 2004). Still, the actual measurement showed that the portal vein pressure after PHx was only twice as much as that before operation (Xie et al., 2014). The increase of intravascular mechanical stress and turbulence promotes the activation of uPA in endothelial cells and other cells. This change regulates the production and release of HGF through β-1 integrin and vascular endothelial growth factor receptor 3 (VEGFR3) pathway. In addition, the permeability of the LSEC fenestra is heighten, and the nitric oxide secretion makes hepatocytes sensitive to HGF (Poisson et al., 2017).

### HGF/C-Met Activates Multiple Signal Cascades in Liver Regeneration

**FIGURE 2:**
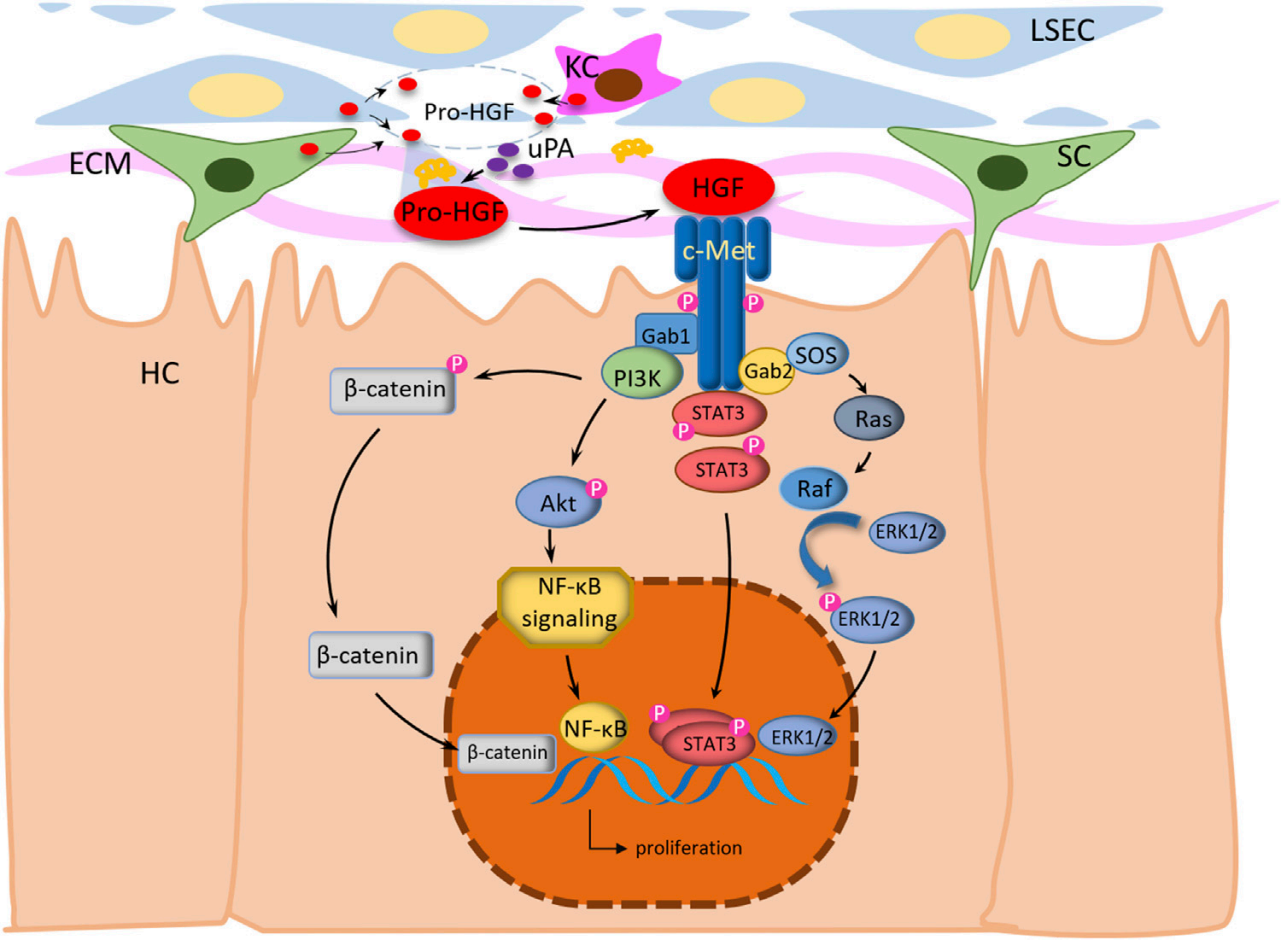
Molecular mechanism of HGF regulating liver regeneration after PHx. HGF is secreted by hepatic stellate cells (HSC), vascular endothelial cells (LECs) and Kupffer cells (KCs). Generally, HGF is sequestered by heparinlike proteoglycans in its inactive form and distributing widely in the extracellular matrix of most tissues. After PHx, inactive Pro-HGF is stored in the liver extracellular matrix (ECM) and can be activated by urokinase-type plasminogen activator (uPA). The activated HGF binding to the c-Met drives the phosphorylation of tyrosine residues and induces nuclear translocation of β-catenin in hepatocytes. In response to c-Met stimulation and the ensuing activation of PI3K, NF-κB signaling is activated, resulting in the nuclear translocation of NF-κB and transcription. Signal transducer and activator of transcription 3 (STAT3) monomers bind to c-Met and become *trans*-phosphorylated, followed by homodimerization and translocation into the nucleus for operate as a transcription factor. Meanwhile, the HGF/c-Met signaling activates Ras by the GRB2-SOS complex, then activates Raf to phosphorylate ERK1/2. Thus, HGF/c-Met signaling is transferred from extracellular to nucleus to activate transcription of a variety of transcription factors essential for liver regeneration.

HGF/c-Met axis has multiple effects in different cell types (Figure 2). The binding of C-Met to HGF initiates the receptor’s innate kinase activity, followed by the formation of a multisubstrate docking site for the intracellular junction protein, which recruits signaling molecules due to homologous dimerization and autophosphorylation at Tyr1234 and Tyr1235 and subsequent phosphorylation at Tyr1349 and Tyr1356 at the c-terminal tail (Birchmeier et al., 2003; Trusolino et al., 2010). Gab1 in the Gab family of docking proteins which can directly or indirectly bind to c-Met through Grb2, is the most critical downstream multi-adapter protein of c-Met (Birchmeier et al., 1997; Nguyen et al., 1997; Lock et al., 2002). The phosphorylated Gab1 creates additional binding sites for most downstream signaling molecules, which activate the different signaling pathways, including JAK/STAT3, phosphoinositide 3-kinase (PI3K)/Akt/NF-κB, Ras/Raf, and mitogen-activated protein kinase (MAPK) cascades.

Because of its rapid response to PHx, STAT3 is considered to be a trigger for liver regeneration. In addition, HGF promotes the phosphorylation of STAT3 to activate the STAT3 pathway (Li et al., 2018b). In mice, the activity of STAT3 DNA binding was observed within 30 min and peaked 3 h after PHx (Cressman et al., 1995). And this response is IL-6-dependent, and this STAT3 activation is almost inhibited in IL-6 deficient mice (Cressman et al., 1996). The STAT3 pathway regulates hepatocyte proliferation through holding cyclin D1/p21 and prevents cell death by up-regulating FLIP, Bcl-2, Bcl-xL, Ref1, and MnSOD (Fujiyoshi and Ozaki, 2011).

The PI3K/Akt pathway plays a vital role in regulating cell growth, migration, differentiation, and apoptosis (Vanhaesebroeck et al., 1999). When c-Met is activated by HGF, PI3K catalyzes the production of phosphatidylinositol-3,4,5-triphosphate after Gab1 coupled to the p85 subunit, which ultimately leads to phosphorylation of c-Met (Sheng et al., 2003; Watanabe et al., 2005).

In the Ras pathway, Erk1/2 as two mitogen-activated protein kinases to transmit mitotic signals, and the HGF/c-Met signaling pathway phosphorylates and activates Erk1/2 after PHx (Spector et al., 1997; Borowiak et al., 2004). Borowiak et al., 2004) found that when the liver regeneration was impaired, the phosphorylation of Erk1/2 was not detected in c-Met mutant mice. The results showed that specific signaling molecules, such as the protein kinase Akt, were fully activated by c-Met and other signaling receptors cooperators. The activation of ERK1/2 kinase was utterly dependent on c-Met during liver regeneration.

The MAPK signaling is activated in response to growth factors and cytokines to promote liver regeneration effectively and plays a dominant role in cellular responses, including proliferation, migration, and differentiation (Pagès et al., 1993; Cowley et al., 1994). HGF in PHx activates MAPK signaling to promote liver regeneration effectively (Xue et al., 2003). In addition, studies have shown that during the adult liver progenitor cells (HPC)/oval cells involved in the repair of chronically injured livers, the HGF/c-Met pathway plays a crucial contribution in regulating TGF-β-mediated epithelial-mesenchymal transition (EMT) responses. The coordination and balance of TGF-β and HGF promoted the optimal regenerative potential of HPCs (Almalé et al., 2019). These data indicate that the HGF/c-Met signaling system is essential for the regeneration of mature organs.

### Regulation of HGF/C-Met Axis

The HGF/c-Met axis is strictly controlled by various mechanisms in normal tissues, and regulated by the amount of active HGF accurately. Two HGF protease inhibitors have been identified, HGF activator inhibitor (HAI)-1 and/or HAI-2, which control the activation of HGF ligand to limit the production of active HGF (Kaido et al., 2004; Kawaguchi and Kataoka, 2014). The HGF-specific antagonist NK4, which is similar to HGF, can bind to C-Met and competitively antagonize downstream phosphorylation induced by HGF (Date et al., 1998; Mizuno and Nakamura, 2013). In addition, the HGF/c-Met axis is regulated by the phosphorylation of the c-Met receptor. Many studies have shown that multiple pathways can negatively regulate the kinase activity of the c-Met receptor. For example, various protein tyrosine phosphatases negatively regulate the c-Met docking site by dephosphorylating c-Met receptor tyrosine residues (Trusolino et al., 2010). And c-Met monoubiquitination, internalization, and degradation could be triggered by casitas B lineage lymphoma ubiquitin ligase. Other studies have shown that changes in certain factors can regulate the expression of HGF and c-Met to regulate the HGF/c-Met axis. *In vitro*, the expression of HGF in hepatic stellate cells underwent a dramatic decrease under hypoxia, and the same as c-Met in hepatocytes (Corpechot et al., 2002). Human placental hydrolysate (hPH) treatment increased the secretion of HGF and promoted the proliferation of hepatocytes in rats after PHx (Lee et al., 2019). Gui et al. (2011) found that cytokine signaling inhibitory factor 1 (SOCS1) regulates hepatocyte proliferation through the HGF/c-Met axis. HGF stimulation-induced c-Met and Gab1 phosphorylation, cell migration, and proliferation in SOCS1 deficient mice hepatocytes. Later studies further showed that SOCS1 not only attenuates HGF/c-Met signaling but also regulates the expression of c-Met (Gui et al., 2015). In the model of chronic liver regeneration in rodents, Annalisa Addante et al. found that the bone morphogenetic protein (BMP)9 and HGF/c-Met signaling axes establish a signal crossover through ALK1 by modulating SMAD1 (pro-survival) and p38 mitogen-activated oval in kinase (p38MAPK; pro-apoptotic) determines the fate of oval cells (Addante et al., 2020). Another study showed that, the cellular transcription factor late SV40 factor (LSF) which is overexpressed in most of human hepatocellular carcinoma cases, is transcribed up-regulated osteopontin (OPN). Then, c-Met is activated *via* the potential interaction between OPN and its cell surface receptor CD44 (Yoo et al., 2011).

Under pathological conditions, many factors regulate cell proliferation by controlling the HGF/c-Met axis. Well known, the liver plays a vital and irreplaceable role in regulating physiological and biochemical balance. It is straightforward to cause pathological liver damage due to various factors such as inflammation and viral infection. Shubham et al. (2019) found that compared to ALF patients, in patients with acute chronic liver failure (ACLF), liver endothelial cells display a significant loss of CXC chemokine receptor type 7 DNA binding inhibitor 1 (CXCR7-ID1) dependent HGF expression, which correlates with the poor hepatocyte proliferation. Research proves that bacterial hepatocyte growth factor receptor agonist InlB321/15, a virulence factor produced by the pathogenic bacterium *Listeria* monocytogenes, improved hepatocyte proliferation and stimulated liver regeneration in animals with 70% hepatectomy (Kalinin et al., 2021).

In addition, some drugs, such as rosiglitazone and GW9662, the peroxisome proliferator-activated receptor gamma (PPARγ) agonist and antagonist, respectively, also regulate cell proliferation through the HGF/c-Met axis. Cheng et al. found that, PPARγ, a nuclear transcription factor with multiple biological functions, plays a important role in regulating cell cycle in liver regeneration (Cheng et al., 2018). They found that rosiglitazone inhibited liver regeneration while and GW9662 accelerated liver regeneration. Blocking the phosphorylation of c-Met by its inhibitor SGX523 could significantly eliminate the enhanced effect of PPARγ antagonist GW9662 on liver regeneration. Activation of HGF/c-Met pathways by phosphorylation of c-Met and ERK1/2 were inhibited in rosiglitazone-treated mice. It indicated that PPARγ hinders liver growth and hepatocyte proliferation by inhibiting the HGF/c-Met/ERK1/2 pathway. Researchers believe that these pathways may represent potential targets for liver disease in the future (Cheng et al., 2018). Schisandra Chinensis and Resina Draconis treatment significantly increased the expression levels of HGF, then promoted the recovery of liver function in liver regeneration model mice through the HGF/c-Met signaling pathway, and ameliorated acute liver injury by promoting liver cell proliferation (Li et al., 2018b; He et al., 2020). Another study showed that rapamycin could promote lens epithelial cells apoptosis by inhibiting HGF-induced phosphorylation of AKT/mTOR, ERK, and JAK2/STAT3 signaling molecules (Tian et al., 2014). At present, a synthetic small molecule, 1K1 has the effect of anti-fibrotic and promoting liver regeneration in rodents. It is an engineered form of naturally occurring HGF fragment NK1, which has the advantages of better stability and is easier to be produced and might be used as a therapeutic medicine for acute and chronic liver disease (Ross et al., 2012). Furthermore, age also plays a role in regulating HGF. It is well known that liver regeneration capacity will decrease with increasing age. Zhu et al. (2014) assessed 130 patients who underwent hepatectomy and found that HGF and c-Met expression in older patients was significantly lower than in younger patients. The average liver volume increase of young patients after 6 months of PHx is substantially more significant than that of elderly patients.

In recent years, there have been many reports on the roles of non-coding RNA (ncRNA) in regulating the HGF/c-Met axis, which are mainly focused on tumor-related studies. C-Met is the target gene of multiple miRNAs. These miRNAs mainly include the miR-34 family (including miR-34a, miR-34b, and miR-34c), miR-206, miR-1, and other tumor suppressor miRNAs. They inhibit cell cycle progression, epithelial-mesenchymal transition, metastasis, stem cell transformation, and angiogenesis and promote cell apoptosis by inhibiting the HGF/c-Met axis (Md Yusof et al., 2020). On the contrary, most lncRNAs targeting c-Met compete for endogenous RNAs (ceRNAs), decoys, or sponges, and combine with specific miRNAs to prevent the inhibition of c-Met, including ceRNAs: lnc-XIST, lnc-MALAT1, lnc-GAPLINC, lnc-HOTAIR targeting miR-34 (Md Yusof et al., 2020), LINC00240 targeting miR-4465 (Cui et al., 2019), LNCRNA ZFAS1 targeting miR-193a-3p (Bu et al., 2020), lncRNA UCA1 targeting miR-1271-5p (Yang and Chen, 2019), etc. However, the research on the mechanisms of ncRNAs in regulating the HGF/c-Met axis in the background of liver regeneration is still minimal. Recent studies have shown that HGF can up-regulate the expression of SNHG12 in regular liver cell lines and activate the Wnt/β-catenin signal pathway to promote hepatocyte proliferation and liver regeneration (Zhu et al., 2020).

### The Roles of HGF/c-Met in Human Liver Regeneration

In human liver regeneration, limited by the means and purposes of clinical treatment, the researches on HGF are mainly carried out in the context of living donor liver transplantation rather than liver resection (Hoffmann et al., 2020). Studies have shown that HGF levels are significantly increased on days 1, 7, and 14 after living donor liver surgery and are related to the recipient liver volume on day 14 (Miki et al., 1996; de Jong et al., 2001; Dłuzniewska et al., 2002; Ninomiya et al., 2003; Efimova et al., 2005; Yoon et al., 2006; Justinger et al., 2008; Matsumoto et al., 2013; Sasturkar et al., 2016; Ray et al., 2018; Sparrelid et al., 2018). Matsumoto et al. (2013) reported that early-phase elevation of serum levels of various growth factors, including HGF, might be associated with the initiation of liver regeneration after hepatectomy in humans. At the same time, Tomiya et al. reported that serum HGF levels were increased after PHx, which was associated with hepatocellular dysfunction, necrosis, and systemic inflammation (Tomiya et al., 1992). On the other hand, Takeuchi et al. (1997) proved that the post hepatectomy bile HGF level might be more meaningful for the early assessment of post hepatectomy liver function, bile HGF level rather than serum HGF level is more closely related to the post hepatectomy liver failure (PHLF). Hoffmann et al. (2020) also suggested that bile HGF as a potentially functional marker of liver function after hepatectomy can be excreted from the liver at a higher concentration than that in serum (Appasamy et al., 1993; Liu et al., 1994b). Furthermore, Lehwald et al. (2014) showed that after hepatectomy, effective HSC mobilization and homing depends on HGF, and these findings have important implications for potential treatment strategies.

## Discussion

The powerful regeneration potential of the liver is legendary, which is attributed to various precise and complicated network regulations. These factors cooperate to restore the metabolic and synthetic functions accurately and timely after liver injury or resection. HGF plays a vital role in the initial stage of liver repair.

HGF has been confirmed closely relating to liver repair after injury by various causes (chemical poison, infection, ischemia, physical injury or PHx, etc.). In animal experiments, HGF initiates liver regeneration at the beginning of the damage. The plasma HGF concentration changes throughout the repair progress, and HGF is maintained at a high concentration during the continuous regeneration phase until regeneration is complete. In clinical cases, the significant increase in HGF after live donor liver transplantation can be observed and related to the final liver regeneration volume. Without its specific receptor c-Met, HGF cannot play a role in liver regeneration. Furthermore, HGF combines with c-Met to trigger downstream cascade reactions and activate JAK/STAT3, PI3K/Akt/NF-κB, and Ras/Raf pathways, affecting cell proliferation, growth, and survival. The reports on HGF in human liver regeneration are minimal, and the use of HGF for drug development is challenging. Still, the preclinical studies currently have a large amount of data to support its therapeutic effects. Numerous clinical trials using rh-HGF protein or targeting HGF genes for clinical treatment are ongoing, which indicates that HGF may be a promising target for the therapies of human diseases.
